# Vitamin D Deficiency Leads to Poorer Health Outcomes and Greater Length of Stay After Total Knee Arthroplasty and Supplementation Improves Outcomes: A Systematic Review and Meta-Analysis

**DOI:** 10.2106/JBJS.RVW.23.00150

**Published:** 2024-04-04

**Authors:** Kavyesh Vivek, Rayan Kamal, Edward Perera, Chinmay M. Gupte

**Affiliations:** 1 Department of Surgery and Cancer, Imperial College University, London, United Kingdom; 2 Imperial College Healthcare NHS Trust, London, United Kingdom

## Abstract

**Background:**

Vitamin D deficiency is increasingly identified as a predictor of poorer outcomes in musculoskeletal disease affecting as many as 1 in 4 people. This study aimed to evaluate the effect of vitamin D supplementation on outcomes after primary total knee arthroplasty (TKA).

**Methods:**

A targeted search of terms related to vitamin D and TKA outcomes was performed in PubMed, Cochrane Central Register of Controlled Trials, ClinicalTrials.gov, American Academy of Orthopaedic Surgeons, and British Orthopaedic Association databases. The results were analyzed using forest plots with I^2^ heterogeneity statistics and pooled effects with 95% confidence intervals (CIs) and p values. A p < 0.05 was considered statistically significant.

**Results:**

A total of 146,054 patients with 150,107 TKRs were analyzed in 10 studies that complied with the inclusion criteria, of which 3 were suitable for meta-analysis. Of these, 4 of the 10 studies showed that vitamin D deficiency resulted in poorer functional outcome scores (Western Ontario and McMasters Universities Osteoarthritis Index, Knee Society Scoring System, and American Knee Society scores), as well as increased risk of revision surgery, incidence of joint infection, and postoperative stiffness. Meta-analysis of length of hospital stay (LOS) demonstrated a significant increase in LOS in patients with vitamin D deficiency (standardized mean difference, −0.54, 95% CI, −0.69 to −0.38, p < 0.00001). Furthermore, outcomes were improved with vitamin D supplementation in 6 of 10 studies.

**Conclusion:**

Vitamin D deficiency results in poorer outcomes of primary TKA, with improved outcomes after supplementation. Further studies should examine the role of preoperative vitamin D screening and/or perioperative supplementation in primary TKA and standardize outcome measures to assess their effect.

**Level of Evidence:**

Level I/II. See Instructions for Authors for a complete description of levels of evidence.

Vitamin D deficiency is increasingly being recognized, affecting 1 in 8 people worldwide and 1 in 4 within the United Kingdom^1^. One of vitamin D's most important functions is to influence the body's calcium axis, which is crucial for maintaining a healthy bone mineral density^2^. Vitamin D has also been shown to positively affect gene expression of osteoblasts and may perform an immunomodulatory function that influences susceptibility to infection^3^. Thus, vitamin D deficiency may lead to adverse consequences in physiology.

For patients with end-stage knee osteoarthritis, total knee arthroplasty (TKA) is the mainstay of treatment to improve quality of life. It is also indicated in the treatment of inflammatory arthritis and osteonecrosis. In the United Kingdom alone, approximately 80,000 knee replacement procedures are performed annually^4^. In the United States, over 2.2 million hip and knee arthroplasties have been performed between 2012 and 2020^5^. However, almost 20% of patients who undergo TKA report dissatisfaction with the procedure postoperatively, with symptoms and causes that include persistent pain, stiffness, lack of functional restoration, and infection^6^. In addition, previous studies have suggested that vitamin D deficiency may result in worse outcomes in TKA, thus leading to even greater dissatisfaction when coupled with the aforementioned symptoms and causes for post-TKA dissatisfaction. Furthermore, the speculated effect of vitamin D on muscle function alludes to a possible compounding effect during the postoperative recovery period; if vitamin D levels are deficient, it can be speculated muscle recovery may take longer, leading to dissatisfaction^7,8^.

Based on previous literature, it can be speculated inhibited bone mineralization resulting from vitamin D deficiency (osteomalacia) may provide a weak scaffold onto which a knee prosthesis/cement mantle can bind, possibly negatively affecting the outcomes of knee arthroplasty^9^. Furthermore, because of poor bone quality, individuals may be more susceptible to periprosthetic fractures^10^, and there may be an effect on muscle-related functional deficit/myopathic pain postoperatively^11^. A previous systematic review by Kenanidis et al. was unable to demonstrate conclusive results on the benefit of vitamin D supplementation^12^. However, Kenanidis et al's systematic review combined hip and knee arthroplasties; had mostly retrospective studies; and had no review of gray literature^12,13^.

To our knowledge, no systematic review and meta-analysis of the pooled data to determine the outcome solely of TKA in vitamin D–deficient patients and whether correction of such deficiency improves outcomes has been conducted.

## Aims and Hypothesis

This study aimed to conduct a systematic review and, where appropriate, a meta-analysis of the literature that evaluated outcomes pertaining to vitamin D insufficiency and supplementation in TKA. The primary outcome measures were functional outcome scores, infection, risk of revision, and length of stay (LOS), with secondary outcomes including stiffness and postoperative pain.

## Methods

### Search Strategy

**Fig. 1:**
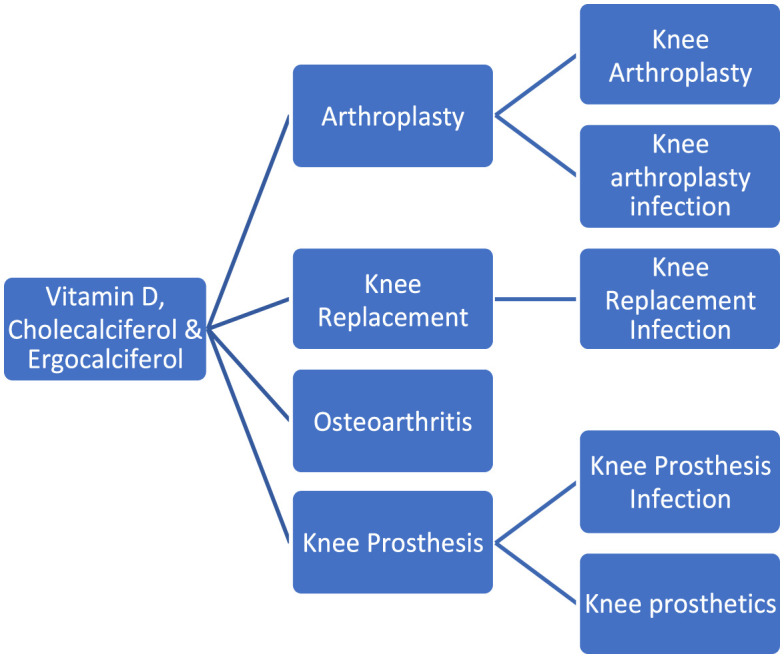
Flowchart to show the different combinations of search terms used.

PubMed, Cochrane Central Library, ClinicalTrials.gov, and gray literature databases were searched. Figure 1 illustrates the combination of search terms used to conduct the systematic search. After duplicate removal, the remaining literature underwent independent title, abstract, and subsequent full-text screening by 2 authors (KV and RK) for eligibility. Disagreement was resolved by consensus or evaluation by the senior authors (EP and CG). If studies met all the inclusion criteria, they were included in the study. Studies were not blinded regarding their source, affiliation, and funding.

### Inclusion and Exclusion Criteria

The inclusion criteria for the study were (1) either randomized control trials or observational studies, (2) adults who received primary knee arthroplasty, (3) surgeries conducted as either an inpatient or outpatient, and (4) articles that have been published in peer-reviewed scientific journals in English.

The exclusion criteria were (1) systematic reviews, (2) meta-analyses, (3) veterinary studies, (4) studies with no explicit connection between vitamin D and knee arthroplasty, and (5) studies with revision TKA as the reported operation.

### Outcome Measures and Data Extraction

Data were extracted by 2 authors (KV and RK) using a data extraction spreadsheet (Microsoft Excel, version 15.2; Microsoft), according to first author, journal name, publication year, Oxford Centre for Evidence-Based Medicine (OCEBM) evidence level, control cohort size number, trial cohort size number, LOS, conclusions, and blood tests^14^.

Primary outcome measures were objective knee scores, patient-reported outcome measures (PROMs), physical function tests, LOS, and risk of infection and revision. Secondary outcome measures were vascular accidents, specifically cerebrovascular accident, myocardial infarction, and deep venous thrombosis (DVT), and postoperative cerebral dysfunction (POCD).

### Statistical Analysis

Formal meta-analyses using random-effects models were conducted, using standardized mean differences to analyze continuous outcomes. Meta-analysis was conducted using RevMan version 5.4. In cases where conducting a meta-analysis was feasible, forest plots were examined, incorporating I^2^ heterogeneity statistics, pooled effect sizes with 95% confidence intervals (CIs), and associated p values. A p < 0.05 was considered statistically significant.

Conclusions were categorized into positive, negative, and neutral. Positive conclusions were defined as marked or statistically significant improvements in study outcome measures between the control and vitamin supplementation groups. Negative conclusions were defined as marked or statistically significant deterioration in study outcome measures between the control and vitamin supplementation groups. Neutral conclusions were defined as no marked or statistically significant differences in study outcome measures between the control and vitamin supplementation groups.

## Results

### Studies

**Fig. 2:**
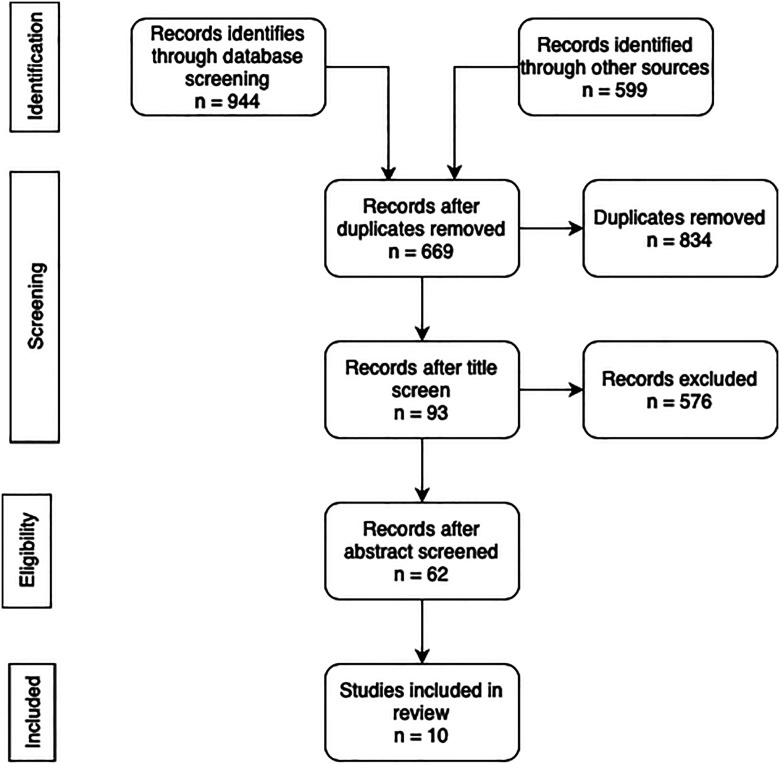
PRISMA flow diagram^15^. PRISMA = Preferred Reporting Items for Systematic Reviews and Meta-Analyses.

A total of 1,503 studies were identified in the primary database search. After duplicate removal, 669 studies remained that were suitable for subsequent screening (Fig. 2). After the screening, 10 studies were relevant to be included in the final systematic review. General reporting on patient demographics was poor throughout the studies, 30% reported mean age, 60% reported length of follow-up of the participants, and 40% reported gender differences. Of the 10 studies, meta-analysis was conducted in 3 of the studies and the other 7 studies were discussed narratively. Twenty-seven CENTRAL trials were excluded because of inaccessible full-text manuscripts, unpublished raw data, not yet commenced study, or just the study protocol being published with no results (Table I).

**TABLE I:** Study Characteristics*

Study	Mean Age (Vitamin D) (yr)	Mean Age (Control) (yr)	Mean Age (yr)	% Male (Vitamin D)	% Male (Control)	% Male	Length of Follow-up (yr)	Sample Size	No. of TKA
Maniar et al., 2016^16^	69	67	N/A	16.07%	21.88%	N/A	0.25	120	120
Lee and Lee, 2015^17^	N/A	N/A	N/A	N/A	N/A	N/A	0.25	191	214
Jansen et al., 2017^18^	N/A	N/A	N/A	N/A	N/A	N/A	8	138	138
Maier et al., 2016^19^	75.5	76.9	N/A	N/A	N/A	47.7	N/A	1,083	477
Hegde et al., 2018^8^	N/A	N/A	N/A	N/A	N/A	N/A	N/A	6,593	6,593
Kong et al., 2020^20^	N/A	N/A	68.8	N/A	N/A	23.2	1	142,147	142,147
Shin et al., 2016^21^	72.4	70.7	N/A	11.40	9.30	N/A	N/A	92	92
Kelly et al., 2017^22^	N/A	N/A	64.9	N/A	N/A	0	N/A	164	164
Gao et al., 2018^23^	N/A	N/A	N/A	N/A	N/A	N/A	7 days	257	129
Reid et al., 2011^24^	N/A	N/A	72	N/A	N/A	42.4	0.25	33	33

* N/A = not available.

**Fig. 3:**
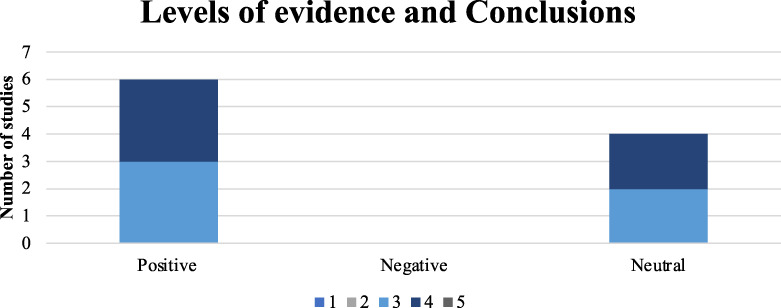
Bar graph illustrating whether the conclusions of each of the 10 studies (n = 10 from PubMed) were positive, negative, or neutral, indicating whether vitamin D supplementation produced beneficial, equivalent, or worse outcomes for patients undergoing total knee arthroplasty.

54.5% were classified as OCEBM evidence level 4 or below, and 60% had positive conclusions associated with vitamin D in TKA, as defined in the Methods section^14^ (Fig. 3).

### Outcomes

**Fig. 4:**
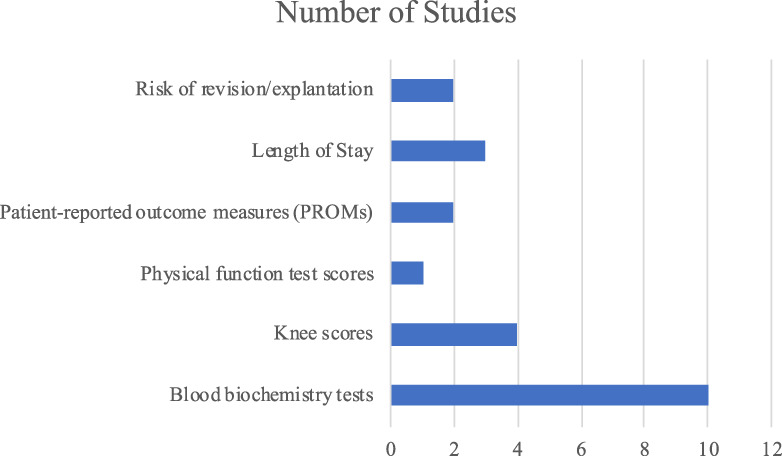
Bar graph illustrating the outcomes measured in 10 studies (n = 10 from PubMed).

Figure 4 outlines the number of studies that report each outcome measure. A total of 4 studies reported that vitamin D sufficiency/supplementation improved outcomes while another 5 studies reported adverse effects associated with vitamin D deficiency (Table II). The study by Hegde et al. reported both positive effects with vitamin D supplementation and complications with vitamin D deficiency^8^. Findings from 8 of the 10 studies concluded that there was an overall positive effect on patient outcomes with the supplementation of vitamin D in TKA (a total of 149,337 patients) (Table II).

**TABLE II:** A Summary Table of 10 Studies (n = 10 from PubMed)*†

Study	Levels of Evidence	Country of Study	Conclusion summary
Kong et al., 2021^20^	4	United States	Cosupplementation of vitamin D and calcium for a greater than 12-month period postoperatively results in reduced risk of revision (p < 0.01) and increased cumulative survival with/without infection
Hegde et al., 2018^8^	3	United States	Vitamin D supplementation resulted in a significant reduction in the risk of prosthesis explantation, surgical site infection requiring debridement and irrigation, stiffness requiring manipulation under anesthesia, DVT, MI, and CVA
Gao et al., 2018^23^	3	United States	25-OH vitamin D was lower in those with POCD, and after both univariate and multivariate analyses, vitamin D levels were identified as the only modifiable risk factor
Jansen et al., 2017^18^	3	Belgium	Vitamin D sufficiency was found to statistically reduce both postoperative length of stay and postoperative WOMAC scores up to 8 yr after TKA.
Kelly et al., 2017^22^	3	Ireland	Vitamin D decreased after TKA in the Irish population
Maniar et al., 2016^16^	4	United States	In patients sufficient in vitamin D, their preoperative WOMAC scores were statistically lower and their postoperative WOMAC scores were clinically lower
Maier et al., 2016^19^	3	Italy	Patients with vitamin D levels >20 ng/L benefitted from a shorter postoperative length of stay
Shin et al., 2017^21^	4	United Kingdom	Postoperative functional KSS, AST, and SMT were all statistically improved. Postoperative clinical KSS, STS, and TUGT showed no statistically significant improvement
Lee and Lee, 2015^17^	4	China	Postoperative WOMAC scores decreased in those with vitamin D levels >30 nmol/L. In those with <30 nmol/L, postoperative WOMAC scores for stiffness and function increased. However, postoperative WOMAC scores for pain decreased for both groups
Reid et al., 2011^24^	4	United States	After a significant systemic inflammatory response, such as in TKA, plasma 25(OH)D is unlikely to be a reliable measure of vitamin D status

* AST = alternative step test, CVA = cerebrovascular accident, DVT = deep vein thrombosis, KSS = Knee Society Score, MI = myocardial infarction, POCD = postoperative cognitive dysfunction, SMT = six-meter test, STS = sit-to-stand test, TKA = total knee arthroplasty, TUGT = timed up and go test, and WOMAC = Western Ontario and McMasters Universities Osteoarthritis Index.

† The level of evidence as defined by the Oxford Centre for Evidence-Based Medicine is simplified to levels 1 to 5^14^.

### Primary Outcome Measures

#### Objective Knee Scores and Patient-Reported Outcome Measures

Four studies reported objective knee scores, including Western Ontario and McMasters Universities Osteoarthritis Index (WOMAC)^25^, Knee Society Scoring system (KSS), American Knee Society (A-KSS) scores, and 1 study using another knee score^26^.

Two further studies^16,17^ reported PROMs (regular medications, physical fitness, pain, EuroQol 5-dimension test^27^, visual analog score, and Quality of Recovery questionnaire^28^). Maniar et al. reported no significant difference in PROMs between the control and study groups. Lee et al. reported that hypovitaminosis D correlated with moderate-to-severe persistent pain^17^.

Maniar et al. demonstrated that preoperative WOMAC scores were statistically lower in those who were vitamin D sufficient, 48.3 compared with 42.3 (p = 0.040). In the postoperative phase, the total WOMAC scores between sufficient and deficient groups had a difference of 1.8 (p = 0.362). This equated to a 4.2-point difference (p = 0.362) in change in WOMAC score after TKA between those with vitamin D sufficiency (30.7 points = 63.6% decrease) compared with those with vitamin D deficiency (26.5 points = 62.6% decrease)^16^.

Shin et al. concluded that functional outcomes (functional A-KSS score, alternative step test [AST], six-meter walk test [SMT], sit-to-stand test [STS], and timed up and go test [TUGT]) after TKAs were adversely affected because of vitamin D deficiency and that surgeons may find benefit in confirming vitamin D levels before TKA to further improve postoperative function. They also reported that the functional A-KSS score, AST, and SMT were all significantly improved, but that clinical KSS score, STS, and TUGT showed no significant changes^21^.

Lee et al. reported a 164% increase in the odds of moderate-to-severe persistent pain associated with vitamin D deficiency. They demonstrated that TKA markedly decreased postoperative WOMAC scores in all study groups, except in the vitamin D–deficient group (≤30 nmol/L vitamin D). Although Lee et al. could not confirm the effect of vitamin D deficiency on the WOMAC score, they demonstrated that stiffness and functional WOMAC scores increased postoperatively in the vitamin D–deficient group^17^.

Jansen et al. demonstrated that those subject to vitamin D deficiency (≤40 mmol/L) had on average a 5-point increase in the WOMAC score over 8 years (p = 0.04)^18^.

Hegde et al. demonstrated, in patients with vitamin D deficiency, there is a 66% increase in the odds of postoperative stiffness requiring manipulation under anesthesia at 3 months and a 69% increase at 1 year (p < 0.001)^8^.

#### Risk of Revision Surgery and Implant Survival

By far, the largest study was by Kong et al. (142,147 patients). It showed that combined vitamin D and calcium supplementation generates a significant reduction of revision surgery risk in both the presence and absence of joint infection at ≥12 months (adjusted hazard ratio [aHR] = 0.63, p = 0.03 and aHR = 0.7, p = 0.008, respectively). Kaplan-Meier analysis demonstrated an increased survival probability in infected (75.25%-93.3%) and uninfected (99.2%-96.67%) TKA patients supplemented with vitamin D and calcium. This correlated with a significant reduction in the implant failure rate at 5 years of 67.1% (72.9% infected, 58.8% aseptic, p < 0.001). Implant survival showed no difference between vitamin D supplementation above or below 800 international units^20^.

Hegde et al. conveyed that the odds of prosthesis revision/explantation were 197% higher in the presence of vitamin D deficiency (p < 0.001), confirming the role of vitamin D deficiency and supplementation in reducing the revision rate^8^.

#### Surgical Site Infection

Hegde et al. concluded that in vitamin D deficiency, there was a 110% increase in the odds of surgical site infection requiring irrigation and debridement at 3 months and a 76% increase at 1 year (p < 0.001 at 3 months and p = 0.001 at 1 year)^8^.

#### Length of Stay

**Fig. 5:**
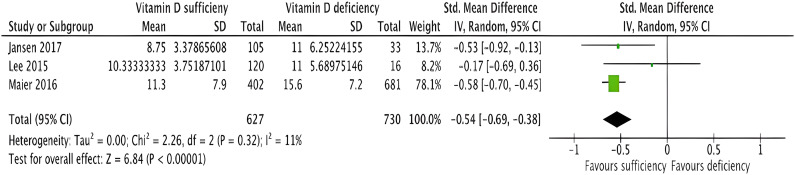
Forest plot to show the effect of vitamin D supplementation on length of hospital stay after knee arthroplasty surgery in 3 studies, compared with placebo supplementation. A spreadsheet calculator from Wan et al. was used to calculate the mean and SD from the sample size and median, range, and/or interquartile range when appropriate for the data available in studies^29^.

Three studies investigated the postoperative LOS (1,357 patients) (Fig. 5). A meta-analysis demonstrated that the LOS was shorter in the vitamin D–supplemented patients compared with the vitamin D–deficient cohort (Fig. 5). Mean LOS was 10.9 days for supplemented vs. 14.6 days for deficient (standardized mean difference, −0.54, 95% CI, −0.69 to −0.38, p < 0.00001) and minimal heterogeneity (I^2^ = 11%).

### Secondary Outcome Measures

Hegde et al. demonstrated a significant increase in the odds of vascular complications, in particular, the odds of deep venous thrombosis (80%, p < 0.001), myocardial infarction (110%, p < 0.001), and cerebrovascular accident (73%, p = 0.006)^8^. Kelly et al. found that post-TKA patients had a mean reduction of 12.5 ± 9.43 nmol/L in vitamin D levels (p = 0.0001). Patients supplemented with vitamin D maintained their vitamin D levels postoperatively significantly better than those who were not supplemented significantly (odds ratio = 6.0985, p = 0.0005)^22^.

Gao et al. demonstrated that preoperative vitamin D levels may be used as a metric to identify the risk of POCD with statistically significantly lower levels in those with POCD. Univariate (p = 0.009) and multivariate (p = 0.016) analyses demonstrated that vitamin D was the only significant modifiable risk factor that improved this outcome^23^ (Table III).

**TABLE III:** A Summary Table of the Associated Benefits of Vitamin D Supplementation Alongside the Associated Complications of Vitamin D Deficiency^16,17,21-23^

Positive Effects of Vitamin D Supplementation	No. of Studies	Complications of Vitamin D Deficiency	No. of Studies
Reduction of hospital stay length	3	Surgical site infection	3
Improvement in outcomes of periprosthetic infection treatment	1	Stiffness requiring manipulation under anesthesia	1
Improvement in postoperative function	2	Prosthesis explantation	1
Reduction in the risk of revision surgery	1	Vascular risk events	1
Improvement in implant survival	1	Pain	1

## Discussion

We aimed to perform a systematic review and meta-analysis comparing the outcomes of primary TKA in vitamin D–sufficient and deficient patients and whether these could be improved by vitamin D supplementation.

Qualitative analysis demonstrated that vitamin D sufficiency resulted in improved functional outcomes after primary TKA compared with those with vitamin D deficiency. Furthermore, vitamin D supplementation improved functional outcomes. Vitamin D status also influenced the incidence of joint infection and risk of revision surgery.

A meta-analysis of studies that fitted the inclusion criteria confirmed that vitamin D deficiency resulted in increased LOS and supplementation reduced LOS after TKA. There was also a reported increase in the incidence of postoperative stiffness, vascular events, and POCD associated with vitamin D deficiency.

A previous systematic review conducted in 2015 on the relationship between vitamin D and surgical outcomes in general found that “hypovitaminosis D is associated with adverse outcomes after diverse surgical procedures.”^30^ A review by Brambilla et al. highlighted the high incidence of vitamin D deficiency present in those undergoing both hip and knee arthroplasties^7^. While suggesting that vitamin D supplementation should be undertaken, they were unable to perform quantitative analysis to justify this because of the availability of literature at the time. Our study is the first to examine the impact of vitamin D status in TKA alone.

### Length of Stay

Our meta-analysis found that vitamin D had a statistically significant impact on reducing LOS. Maier et al. specifically stated that vitamin D supplementation was both a safe and simple measure to reduce LOS^19^. Jansen et al. suggested that vitamin D supplementation may not only improve short-term functional outcomes and reduce LOS but there may also be an unknown impact on long-term post-TKA functional outcomes, which requires further study^18^. Longer LOS increases the chance of further complications such as DVT, stiffness, and noncompliance^31^.

One possible explanation for vitamin D status affecting LOS could be its speculated influence on muscle function. A previous study has identified certain genomic pathways in which vitamin D may play a role in muscle contraction and recovery—both essential for optimal recovery^21^. Another possible explanation may be the influence of vitamin D on pain, anxiety, and depression levels after TKA^32^. However, the definitive reason for why vitamin D status affects LOS still requires further research and specific studies to evaluate these 2 variables as primary outcomes. Furthermore, low vitamin D levels can be used as a prognostic indicator for POCD in TKA patients^23^ because it is more likely that patients with POCD may suffer from a prolonged LOS.

### Postoperative Infection and Risk of Revision

Arshi et al. suggested that vitamin D supplementation is a potentially cost-effective method of reducing prosthetic joint infections in TKA. A predictive cost model suggests that selective screening and replacement potentially saves an estimated $1,504,857 per 10,000 primary TKA surgeries and nonselective replacement saves $1,906,077 per 10,000 primary TKAs, compared with no replacement of vitamin D^33^.

Periprosthetic fragility fractures, associated with vitamin D deficiency, present a significant challenge to the orthopaedic surgeon and the patient, further consolidating the cost-effective nature of vitamin D supplementation^34^. Okafor et al. suggested that revisions in the context of infection may cost as much as 4 times greater than those performed in the absence of infection. A cost-benefit analysis of vitamin D and calcium supplementation calculated a net cost benefit of more than €5.7 billion in the prevention of fragility fractures^35^. In addition, Kong et al. reported that proper bone matrix formation is facilitated by the role that vitamin D plays in calcium absorption and suggests that a weaker implant-to-bone interface is one of the primary causes of arthroplasty revision^20^. The inexpensive and easy supplementation of vitamin D may also help with the reduction of infection because vitamin D regulates macrophages, dendritic cells, and lymphocytes as previously reported by Beard et al.^36^.

### The Role of Preoperative Vitamin D Deficiency Screening and/or Supplementation Perioperatively in Primary TKA

Our study raises the possibility of whether preoperative screening of patients undergoing TKA should include and ideally correct their vitamin D status. This has the potential to not only reduce LOS but also reduce postoperative complications, including stiffness and suboptimal function.

This claim is supported by the fact that vitamin D screening meets all the criteria set out in Wilson and Junger's principle for screening^37^. Low vitamin D levels are important in the context of knee replacement. It is recognizable in both the early and late phases through preoperative assessment and is a condition that is understood to an adequate level. We believe that vitamin D level tests are acceptable to the public and can be easily incorporated into preoperative assessments. Regarding treatment of low vitamin D levels in TKA, vitamin D supplementation is a cost-effective treatment and there is current guidance for levels at which treatment should begin^33^. Nonetheless, no included studies definitively found a specific target dosage for vitamin D supplementation for all cohorts.

However, Arshi et al. suggested that a dedicated screening tool may be more expensive than universal supplementation before TKA, so the practicality and benefit of such a screening tool need to have further research^33^.

Similarly, Zajonz et al. found that to truly assess the impact of vitamin D supplementation in any screening capacity, a vitamin D–binding protein (VDBP) level in serum or genetic testing of the VDBP gene would be required—both tests are very expensive and thus not as cost-effective as supplementation in all patients subject to TKA regardless of preoperative vitamin D status^38^.

### Limitations

Various knee scores have been used to evaluate outcomes in TKA. Our review found that there was no consistent scoring system that was preferred for evaluating outcomes after TKA, which made meta-analysis challenging. Future directions in this area would be toward consensus regarding a combination of currently used standardized metrics, along with questions focused on a patient-specific goal, such as those used in the Patient Activation Measure (PAM) questionnaire^39^.

## Conclusion

Our study is the first systematic review and meta-analysis examining the association of vitamin D deficiency and supplementation on outcomes solely in primary TKA. Although there was heterogeneity of studies, the vast majority reported an increase in adverse outcomes with vitamin D deficiency and improvements in outcomes after supplementation. Our meta-analysis demonstrated a significant reduction in LOS for TKA patients in whom vitamin D status was optimal.

Further studies should examine the role of preoperative screening for vitamin D insufficiency or vitamin D supplementation in all patients incorporated into standard TKA protocols. Standardized assessment scores are also needed to assess the benefits of vitamin D screening and supplementation in TKA.

### Source of Funding

This study has had no funding from any external bodies, and all researchers have carried out this work pro bono.

## Appendix

Supporting material provided by the authors is posted with the online version of this article as a data supplement at jbjs.org (http://links.lww.com/JBJSREV/B72). This content was not copyedited or verified by JBJS.
